# Comparative study of clinical grade human tolerogenic dendritic cells

**DOI:** 10.1186/1479-5876-9-89

**Published:** 2011-06-09

**Authors:** M Naranjo-Gómez, D Raïch-Regué, C Oñate, L Grau-López, C Ramo-Tello, R Pujol-Borrell, E Martínez-Cáceres, Francesc E Borràs

**Affiliations:** 1 Laboratory of Immunobiology for Research and Diagnosis (LIRAD). Blood and Tissue Bank (BTB); Dept. of Cell Biology, Physiology and Immunology, Universitat Autònoma de Barcelona, Institut Investigació Germans Trias i Pujol, Spain; 2 Multiple Sclerosis Unit. Department of Neurosciences, Hospital Universitari Germans Trias i Pujol Badalona Barcelona. Spain

## Abstract

**Background:**

The use of tolerogenic DCs is a promising therapeutic strategy for transplantation and autoimmune disorders. Immunomodulatory DCs are primarily generated from monocytes (MDDCs) for *in vitro *experiments following protocols that fail to fulfil the strict regulatory rules of clinically applicable products. Here, we compared the efficacy of three different tolerance-inducing agents, dexamethasone, rapamycin and vitamin D3, on DC biology using GMP (*Good Manufacturing Practice*) or clinical grade reagents with the aim of defining their use for human cell therapy.

**Methods:**

Tolerogenic MDDCs were generated by adding tolerogenic agents prior to the induction of maturation using TNF-α, IL-β and PGE2. We evaluated the effects of each agent on viability, efficiency of differentiation, phenotype, cytokine secretion and stability, the stimulatory capacity of tol-DCs and the T-cell profiles induced.

**Results:**

Differences relevant to therapeutic applicability were observed with the cellular products that were obtained. VitD3-induced tol-DCs exhibited a slightly reduced viability and yield compared to Dexa-and Rapa-tol-DCs. Phenotypically, while Dexa-and VitD3-tol-DCs were similar to immature DCs, Rapa-tol-DCs were not distinguishable from mature DCs. In addition, only Dexa-and moderately VitD3-tol-DCs exhibited IL-10 production. Interestingly, in all cases, the cytokine secretion profiles of tol-DCs were not modified by a subsequent TLR stimulation with LPS, indicating that all products had stable phenotypes. Functionally, clearly reduced alloantigen T cell proliferation was induced by tol-DCs obtained using any of these agent. Also, total interferon-gamma (IFN-γ) secretion by T cells stimulated with allogeneic tol-DCs was reduced in all three cases, but only T cells co-cultured with Rapa-tol-DCs showed impaired intracellular IFN-γ production. In addition, Rapa-DCs promoted CD4+ CD127 low/negative CD25high and Foxp3+ T cells.

**Conclusions:**

Our results demonstrate contrasting influences of different clinical-grade pharmacological agents on human tol-DC generation. This should be taken into account for decisions on the use of a specific agent for the appropriate cellular therapy in the context of a particular disease.

## Background

Autoimmune diseases are characterized by the loss of tolerance toward self-antigens and the induction of destructive immune responses leading to tissue damage. Most patients with autoimmune diseases are treated with immunosuppressive drugs that induce a generalized immune suppression, which increases the risk of infectious diseases and cancer [1]. Thus, induction of tolerance is an important goal for treating autoimmune disorders or to prevent undesirable immune responses against allogeneic transplants [2-8].

Research in recent years has primarily focused on developing more selective immunosuppressive or immunomodulatory therapies with fewer side effects and with the potential for long-term disease remission. In this context, the use of antigen-specific tolerogenic dendritic cells (tol-DCs) that target autoreactive T cells is an attractive strategy, with the aim of reprogramming the immune system for the treatment of autoimmune disorders [9-11].

Dendritic cells (DCs) are professional antigen-presenting cells that have the potential to either stimulate or inhibit immune responses [12-15]. Their broad range of powerful immune stimulatory and regulatory functions has placed DCs at centre stage of active immunotherapy [16-23]. Dendritic cells maintain immune tolerance to self-antigens by deleting or controlling the pathogenicity of autoreactive T-cells. Modifications of DCs in the laboratory can enhance and stabilise their tolerogenic properties, and several pharmacological agents, such as dexamethasone (Dexa), rapamycin (Rapa) and vitamin D3 (VitD3), may promote the tolerogenic activities of DCs [24,25]. It has been widely reported that such maturation-resistant DCs can regulate autoreactive or alloreactive T-cell responses and promote or restore antigen-specific tolerance in experimental animal models [26-36].

Yet, the current challenge is to move tol-DCs from the bench to the bedside [37-41], and one of the major tasks is to translate laboratory protocols into clinically-applicable procedures. Currently, information on different tolerogenic cellular products can be found at the research level. Therefore, a systematic comparison of the required functional characteristics of the various clinical tolerogenic DCs is necessary.

In this study, we compared the effects of three immunomodulatory agents: Dexa, Rapa and VitD3, on tol-DCs generation using clinical grade reagents. We describe both the convenient and inconvenient aspects of each different "tolerogenic cellular products" to induce tolerance and discuss the eligibility of each cellular product for particular therapeutic scenarios.

## Methods

### Culture Media and reagents

Culture medium used was X-VIVO 15 (BioWhittaker^®^, Lonza, Belgium) supplemented with 2% (vol/vol) heat-inactivated AB human serum (BioWhittaker^®^, Lonza, Belgium), 2 mM L-glutamine (Sigma-Aldrich Company LTD, Saint Louis, MO, USA), 100 U/mL penicillin (Cepa S.L, Madrid, Spain), and 100 μg/mL streptomycin (Laboratorios Normon S.A, Madrid, Spain).

### Monoclonal Antibodies

The following murine mAbs were used. FITC-labelled mAbs: CD86 and Foxp3 (BD Biosciences, CA, USA); PE-labelled mAbs: CD14 (ImmunoTools GmbH, Germany), CD40 and CD127 (BD Biosciences); PerCP-labelled mAb: CD3 (BD Biosciences); PE-Cyanine dye 5-labelled mAb: CD25 (BD Biosciences); PE-Cyanine dye 7-labelled mAb: CD14 (BD Biosciences); Allophycocyanin (APC)-labelled mAbs: CD83, CD4 and anti-IFN-γ (BD Biosciences); APC-H7-labelled mAb: HLA-DR (BD Biosciences).

### Immunostaining and flow cytometry

Cells were washed, resuspended in 50 μl of PBS and incubated with mAbs for 15-18 minutes at room temperature (RT). After washing, acquisition used a FacsCanto II flow cytometer with Standard FacsDiva software (BD Biosciences). Subsequent analyses used FlowJo software (Tree Star, Inc, OR, USA). Samples were gated using forward (FSC) and side (SSC) scatter to exclude dead cells and debris.

### Cell Isolation

Buffy coats, provided by our Blood Bank department, were obtained from healthy blood donors following the institutional Standard Operating Procedures for blood donation and processing. Peripheral Blood Mononuclear Cells (PBMCs) were isolated by Ficoll-Paque (Lymphoprep, Axis Shield, Oslo, Norway) density gradient centrifugation at 400 × g for 25 min. Recovered cells were washed twice in PBS and counted using Perfect Count microspheres (Cytognos SL, Salamanca, Spain) following the manufacturer's instructions. The Ethical Committee of Germans Trias i Pujol Hospital approved the study, and all subjects gave their informed consent according to the Declaration of Helsinki (BMJ 1991; 302: 1994).

### Establishing Monocyte-derived DCs

PBMCs were depleted of CD3+ T cells using a RosetteSep™ Human CD3 Depletion Cocktail (StemCell Technologies, Seattle, WA, USA). Monocytes were obtained by positive selection using an EasySep^® ^Human CD14 Positive Selection Kit (StemCell Technologies, Seattle, WA, USA). For all samples, the purity and viability of the monocyte populations were greater than 95% and 90% respectively, as assessed by the expression of specific markers and Annexin V + and *7*-Amino-actinomycin D (7AAD) labelling (BD Biosciences).

Monocytes were cultured at 1-1.1 ×10^6^/ml for 6 days in cGMP-grade XVIVO15 containing penicillin (100 U/ml) and streptomycin (100 μg/ml) in the presence of clinical-grade granulocyte-macrophage colony-stimulating factor (GM-CSF: 1000 U/ml; CellGenix, Freiburg, Germany) and interleukin 4 (IL-4: 1000 U/ml; CellGenix, Freiburg, Germany). Cells were replenished on day 2 with a half volume of fresh medium and cytokines, and complete fresh medium and cytokines on day 4. To induce mature DCs (Mat-DCs), DCs were treated with a cGMP-grade cytokines cocktail: TNF-α (1000 U/mL) and IL-β (10 ng/mL) (both from CellGenix); and PGE2 (1 μM) (Pfizer, New York, USA) on day 4. Tol-DCs were established by treatment with either Dexa (1 μM, Fortecortín, Merck Farma y Química, S.L, Spain), Rapa (10 nM, Rapamune, Wyeth Farma S.A, Spain) on days 2 and 4, or VitD3 (1 nM, Calcijex, Abbott) on days 0 and 4. Tol-DCs were stimulated as mature DCs at day 4 with the cytokine cocktail. On day 6, DCs were harvested and washed extensively twice before functional assays were performed.

### Allostimulatory assays

PBMCs were labelled with CFSE and plated (10^5 ^cells/well) in 96-well round-bottom plates. Mononuclear cells were co-cultured for 6 days with MDDCs at a 1:20 ratio (DC: PBMC). Cell proliferation was determined by the sequential loss of CFSE fluorescence of CD3 positive cells, as detected by flow cytometry.

### Intracellular cytokine staining

Mononuclear cells isolated from healthy donors were seeded in 96-well round bottom plates (Nunc) at a density of 1 × 10^5 ^cells/well and stimulated for 6 days with allogeneic DCs (5 × 10^3 ^DC/well). Then, total cells were stimulated with 50 ng/mL phorbol 12-myristate 13-acetate (PMA, Sigma) plus 500 ng/mL ionomycin (Sigma) for 5 h in the presence of 10 μg/ml brefeldin A (Sigma). After stimulation, cells were washed with PBS and stained for 18 min at RT with PerCP-conjugated anti-human CD3 mAb (BD Biosciences). Cells were then washed, fixed and permeabilised using an IntraStain kit (Dako) and incubated for 28 min at RT with anti-human IFNγ APC mAb (eBioscience). Cells were washed and analysed with a BD-FACScanto II flow cytometer equipped with FACSDiva software (Becton-Dickinson).

### Measurements of cytokine production

Interleukin 10 (IL-10), IL-12p70 and IL-23 were determined in supernatants of activated DCs using MILLIPLEX Multi-Analyte Profiling (MAP; Millipore Corporate Headquarters, MA, USA) following the manufacturer's instructions. These supernatants were collected after 48 h upon maturation and also after strong TLR (LPS: 100 ng/mL from E. Coli 0111:B4, Sigma. Reference: L4391) re-stimulation for 24 h and analysed for the presence of the indicated cytokines.

Supernatants from allogeneic co-cultures were collected after 6 days, stored at -20°C, and analyzed by MILLIPLEX Multi-Analyte Profiling (IL-10) and ELISA (TGFβ, eBioscience).

### Determination of CD4+ CD127 low/negative CD25high and Foxp3+ T cells

CD3+ T lymphocytes were purified from mononuclear cells by negative selection using an EasySep^® ^Human T Cell Enrichment Kit (StemCell Technologies) following the manufacturer's instructions. Purity was > 95% in all experiments. Enriched T cells were plated (10^5 ^cells/well) in 96-well round-bottom plates. After 6 days of co-culture (1DC:20T), we used flow cytometry to determine the percentages of Tregs defined as CD4+, CD127^low/negative^, CD25^high ^and intracellular Foxp3+, as previously reported [42] (Human Regulatory T Cell Staining Kit; eBioscience, San Diego, CA, USA).

### Statistical analyses

Results are given as means ± standard deviations (SD) for n samples per group. Results are the means of at least 5 replicates for each experiment. Comparisons used either parametric paired t-tests or non-parametric Wilcoxon tests, as appropriate. A p-value ≤ 0.05 was considered statistically significant. Prism software (GraphPad v4.00 software. CA, USA) was used for statistical analysis.

## Results

### Dexa, Rapa and VitD3 generate tol-DCs under GMP conditions

Most clinical studies use MDDCs to obtain adequate numbers of cells to warrant clinical doses for patients. We first evaluated the viabilities and yields of the differentiation processes using parallel conditions for the same individual for each of 5 different donors. In order to establish a common, objective baseline for comparative purposes, dose-dependent experiments were set up to obtain the optimal concentration of each immunomodulatory agent that induced an arbitrary 50% reduction of allostimulatory capacity compared to mature DCs (similar to immature DCs) with high viability (≥ 85% viable cells) (additional file 1:, Figure S1). Rapa-and VitD3-tol-DCs exhibited 50-70% reductions of T proliferation at 10 nM and 1 nM, respectively, while Dexa required a concentration 100-1000 times higher (1 μM) to achieve similar results. These criteria allowed us to evaluate equivalent tolerogenic products using the following final concentrations: 1 μM Dexa, 10 nM Rapa and 1 nM VitD3.

**Figure 1:**
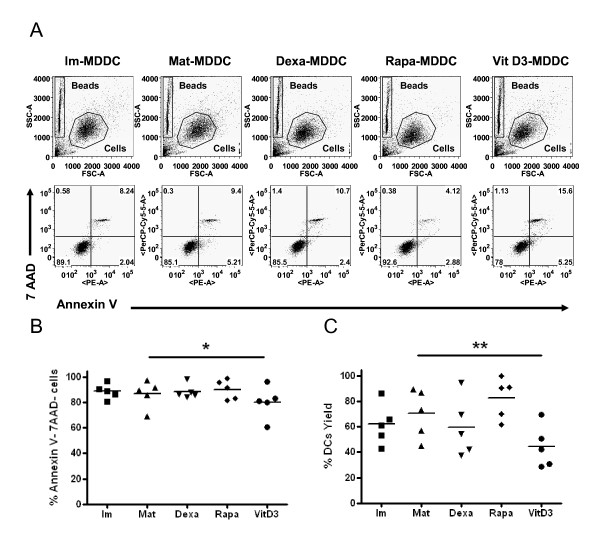
**Survival of tol-DCs after clinical protocol differentiation**. (A) Viability of MDDCs with or without immunomodulatory treatment after 6 days of differentiation. Plots are representative of 5 independent experiments. (B) Surviving cells are annexin V and 7AAD negative cells. (C) Yield obtained calculated as the number of MDDCs obtained from the initial number of monocytes that were cultured (n = 5). (paired t-test. * p ≤ 0.05; ** p ≤ 0.001; ***≤ 0.0001).

Simultaneous staining of cells with PE-annexin V and with the non-vital dye 7AAD was used to discriminate viable cells (Figure 1A). These results showed that, compared to mature DCs, only VitD3 treatment slightly reduced the cell viability (80 ± 13% vs. 87 ± 11% of mature DCs, p = 0.01, paired t-test; Figure 1B) and yield of DCs (45 ± 17% vs. 70 ± 19%, p = 0.0071, paired t-test; Figure 1C) (n = 5). Treatment with Dexa and Rapa did not affect these outcomes (viability: 89 ± 6% and 90 ± 8% and yield: 60 ± 23% and 83 ± 16%; respectively, n = 5).

### Dexa-and Vit D3-tol-DC phenotypes change and produce IL-10

The tolerogenic functions of DCs may depend on their maturation stage and their anti-inflammatory profile. Thus, in our initial studies, we investigated the surface phenotypes and cytokine milieus of tol-DCs obtained using the 3 different immunomodulatory agents.

**Figure 2:**
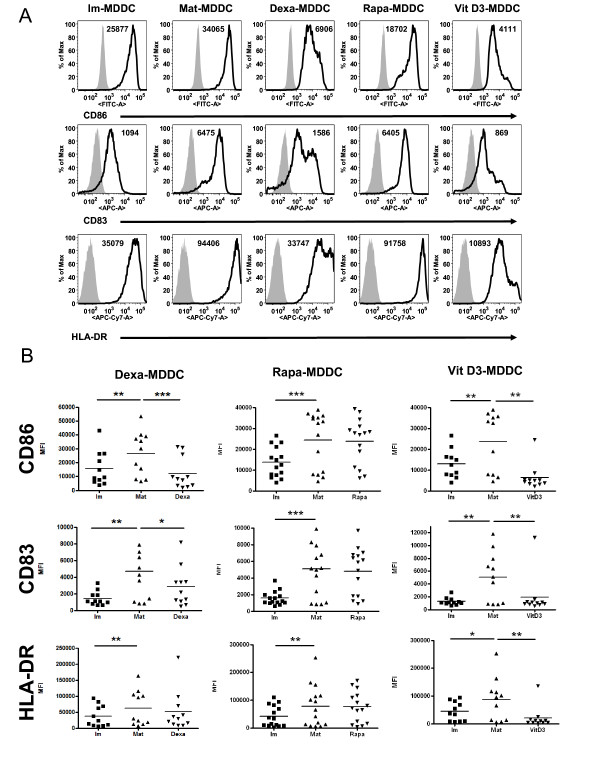
**Dexa-and VitD3-DCs exhibit a semi-mature phenotype as compared with Mat-DCs**. (A) DC expression of maturation-associated markers of immature DCs (Im-DCs), mature DCs (Mat-DCs) and tol-DCs. Surface expression of CD86-FITC, CD83-APC and HLA-DR-APCH7 staining on MDDCs. Each histogram is representative of 15 independent experiments. Isotype controls are shown in grey. (B) Results are mean fluorescence intensities from n = 11 cultures in the presence of Dexa, n = 15 cultures with Rapa-DCs and n = 11 cultures with VitD3-DCs. (paired t-test. * p ≤ 0.05; ** p ≤ 0.001; ***≤ 0.0001).

After 6 days of differentiation, immature DCs (Im-DCs) expressed low surface levels of MHC II and co-stimulatory molecules (CD86 and CD83; n = 15) as compared with mature DCs (Mat-DCs) (Table 1 and Figures 2A and 2B). Tol-DC generation in the presence of Dexa and VitD3 was associated with an immature phenotype as compared to Mat-DCs. This phenotypic impairment may affect the whole population or may be observed as a partial maturation induced in a relatively low proportion of cells compared to the mature situation. The latter was often observed in most cases of our results. Indeed, in several experiments the percentage of cells with low CD83 and HLA DR levels ("semi-mature") was over 75%. As our study aimed for the comparison of the populations obtained under different tolerogenic regimes, we considered that the analyses of the whole population would better reflect these comparisons. VitD3-DCs showed a significantly reduced expression of CD86, CD83 and HLA-DR (n = 11). Dexa-tol-DCs exhibited a similar pattern, although only CD86 and CD83 showed significantly reduced expression levels (n = 11). In contrast, Rapa-tol-DCs were not phenotypically different from Mat-DCs (n = 15) (Table 1 and Figures 2A and 2B).

**Table 1:** Surface markers on tolerogenic DCs

	CD86	CD83	HLA-DR	n
**Im-DC**	15737 ± 7681 ***	1316 ± 673 ***	39405 ± 33712 **	15
**Mat-DC**	22704 ± 13632	4371 ± 3189	70692 ± 66038	15
**Dexa-DC**	12291 ± 11364 ***	2811 ± 2343 *	50928 ± 62830	11
**Rapa-DC**	23782 ± 10961	4785 ± 2786	75297 ± 56014	15
**VitD3-DC**	6398 ± 6243 **	1941 ± 3096 **	20851 ± 38803 **	11

Surface markers expression was measured by flow cytometry on MDDC. Results are the averages ± SDs of Mean Fluorescence Intensity (MFI) from different donors; n (number of samples). Mature DCs were used as a reference group for all comparisons. * p ≤ 0,05; ** p ≤ 0,001; *** p ≤ 0,0001 (paired t-test) indicating significant differences compared to MDDCs.

**Figure 3:**
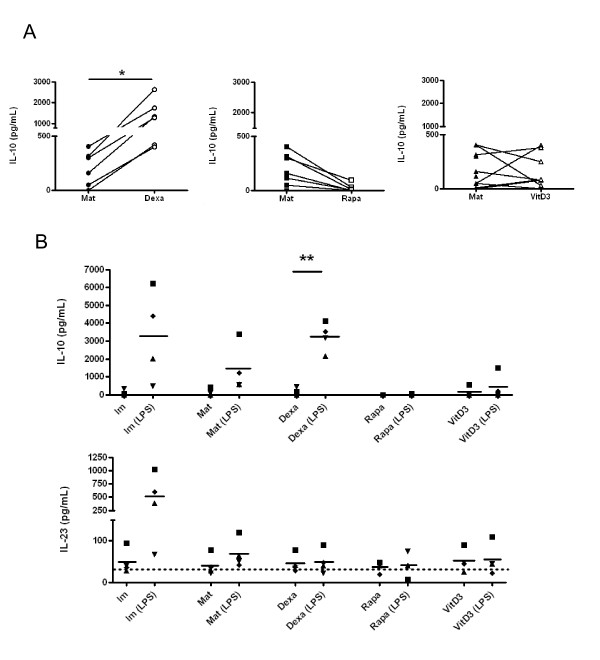
**Tolerogenic dendritic cells (tol-DCs) exhibit an anti-inflammatory cytokine profile and stable phenotype**. (A) IL-10 release by DCs in the presence or absence of immunomodulatory agents (Dexa, Rapa or VitD3) was measured after 48 h stimulation with a maturation cocktail. Supernatants were harvested and analysed for IL-10 production by MILLIPLEX (Dexa: n = 6; Rapa: n = 7 and VitD3: n = 11). (B) Stability of tol-DCs was evaluated after culture for 24 h in XVIVO medium containing LPS (without immunomodulatory agent). IL-10 and IL-23 production was determined for all DC conditions (with or without LPS). (n = 4. Statistical significance derived from a paired t-test. * p ≤ 0.05).

In addition, we measured the secretion of IL-10 and IL-12p70 after 48 h upon maturation. We found IL-10 production in cultures with either Dexa or VitD3, but not with Rapa (Figure 3A). Of note, the production of IL-10 in the presence of dexamethasone was 6 times higher compared to mature DCs (1305 ± 846 pg/mL vs. 204.5 ± 160.5 pg/mL; p = 0.0135, n = 6, paired t-test). Also, VitD3 tol-DCs produced slightly more IL-10 than mature cells (243 ± 272.9 pg/mL vs. 204.5 ± 160.5 pg/mL, n = 11). In contrast, IL-12 was notably undetectable in all culture conditions (data not shown).

### Stability of Tol-DCs after restimulation with LPS

To evaluate whether DCs were resistant to an exogenous maturation stimulus, tol-DC stability was investigated by culturing tol-DCs for 24 h in XVIVO medium containing LPS (without immunomodulatory agent). As shown in Figure 3B, tol-DCs were phenotypically refractory to secondary stimulation, and retained their typical cytokine profile of IL-10 production. Dexa tol-DCs restimulated with LPS produced 19 times more IL-10 than Dexa-DCs (165.1 ± 203.7 pg/mL vs. 3244 ± 828.6 pg/mL, p = 0.0046, n = 4, paired t-test). Regarding VitD3-DCs, LPS-restimulation did not greatly modified the IL-10 production. Again, Rapa tol-DCs did not exhibit any IL-10 production.

Importantly, while primary stimulation of the DCs with this strong TLR4 ligand induced greater IL-23 production by immature DCs (10.86 ± 6.5 fold increase), no increased IL-23 production was detected by tol-DCs in any culture condition (Dexa-DC: 1.11 ± 0.46; Rapa: 1.22 ± 0.84; VitD3: 1.08 ± 0.51 fold changes), which supported a stable non-proinflamatory profile for tol-DCs. Mat-DC also showed some refractoriness to the ulterior stimulation with LPS, meaning there was a faint production of cytokines "de novo" as opposite to Im-DCs.

### DC-tols do not promote a Th1 profile

**Figure 4:**
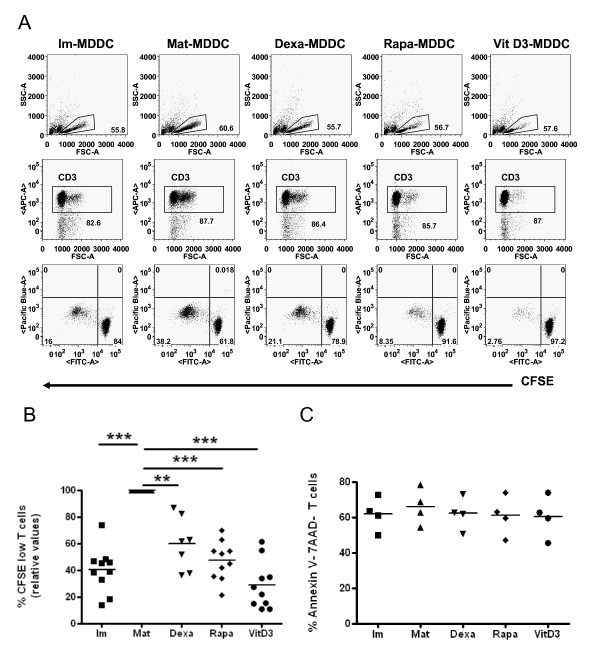
**Tolerogenic dendritic cells (tol-DCs) suppress T cell proliferation without apoptosis induction**. (A and B) Allogeneic T cells were stimulated with tol-DCs and compared for proliferation with stimulation by Mat-DCs and Im-DCs in mixed-lymphocyte reactions. Compared to Mat-DCs, tol-DCs potently inhibited allogeneic T cell proliferation at a level similar to Im-DCs (Dexa: n = 7; Rapa: n = 10; and Vit D3: n = 10). (C) Viability results (%Annexin V and 7AAD negative) for T cells co-cultured with different cellular products (n = 4).

To analyze the effect of the different tol-DCs, allostimulated T cells were further studied. An example of the proliferation of T cells allostimulated by tol-DCs is shown in Figure 4A. We have also summarized the relative results achieved using mature-DCs for different donors in Figure 4B. Of mention, we found that Dexa-DCs inhibited T cell proliferation only partially in some donors (4/12 subjects, data not shown).

To further investigate the effect of tol-DCs on T cells, we also determined whether inhibition of T cell proliferation was due to increased T cell apoptosis. We found that the reduced stimulation of T cell proliferation was not due to a reduction in cell viability induced by a particular type of tol-DC (% of both Annexin V and 7AAD negative cells) of allostimulated T cells (Im: 61.76 ± 9.28%; Mat: 65.92 ± 10.13%; Dexa: 62.08 ± 9.21%; Rapa: 61.02 ± 11.12% and VitD3: 60.43 ± 11.72%; n = 4) (Figure 4C).

**Figure 5:**
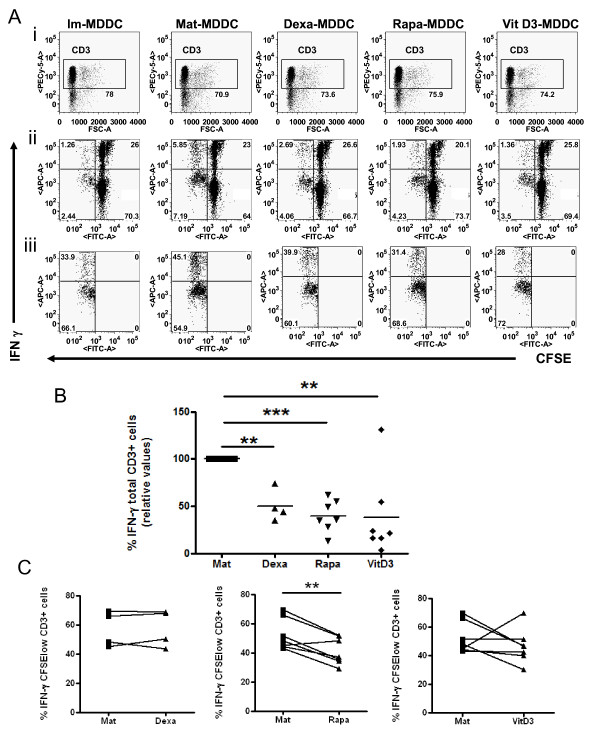
**Decreased production and secretion of IFN-γ by T lymphocytes stimulated with tol-DCs**. Proliferating T lymphocytes were obtained from allostimulatory cultures. The production of interferon (IFN)-γ was measured by intracellular staining after restimulating the cells with PMA+Io in the presence of brefeldin for 5 h. (A) First row (i) shows gating CD3+ cells. The second row plots (ii) indicate the proportion of total IFN-γ producing cells. Third row (iii) shows the percentages of cells that responded to allostimulation (CFSElow) and produced IFN-γ. The numbers inside the plots indicate the percentage of cells in each quadrant or boxes (a representative experiment). (B) Summary of the results of the total intracellular IFN-γ (Upper Left, UL) production with Dexa-(n = 4), Rapa-(n = 7) and Vit D3 (n = 7) activated cultures relative to Mat-DCs (taken as 100% production). (C) Percentage of IFN-γ producing T cells that responded to allostimulation (CFSE^low ^CD3+ cells). Each symbol represents an individual sample. Significant differences are indicated (** p < 0,001; paired t-test).

To gain some insight into the cytokines secreted by these responding T cells, CFSE^low ^alloproliferative T lymphocytes were re-stimulated with PMA + ionomycin and IFN-γ production was measured by intracellular staining. These results confirmed a reduction of about 50-60% in IFN-γ production relative to mature DCs for all conditions tested (Figures 5A and 5B: 50.18 ± 16.65% IFN-γ producing cells among T cells allostimulated by Dexa-DC, p = 0,0093, n = 4, paired t-test; 39.83 ± 16.76% Rapa-DC, p < 0,0001, n = 7, paired t-test; and 37.97 ± 44.08 VitD3-DC, p = 0,0098, n = 7, paired t-test). When only CFSE^low ^proliferating T cells were analysed, Rapa-DCs stimulated T cells showed a significant decrease in IFN-γ production relative to Mat-DCs (Figure 5C: 40.99 ± 9.2% vs. 52.47 ± 10.85% IFN-γ among CFSE^low ^CD3+ cells, n = 7, p = 0,0057, paired t-test). VitD3-DCs also suppressed IFN-γ production in co-cultures with allogeneic mononuclear cells, but only in some donors and Dexa-DCs did not reduce the capability of responding T cells to produce IFN-γ in any of the experiments.

In addition, we determined the production of IL-10 and TGFβ in the supernatants from T cells co-cultured with tol-DC. We could measure IL-10 production in allostimulated T cells by Dexa-DC in 3 of 4 donors. Interleukin 10 values obtained were 57.47 ± 29.47 pg/mL (T cells + Dexa-DCs) compared to 33.37 ± 2.66 pg/mL (T cells allostimulated with Mat-DCs). Conversely, we did not find major differences in T cells stimulated with Rapa-DC (15.7 ± 13.61 pg/mL) or VitD3-DC (38.7 ± 7.28 pg/mL) compared to mature DCs (n = 3). Regarding TGFβ, all the measures were below the limit of detection of the assay (60 pg/mL) in the different stimulatory conditions analyzed.

**Figure 6:**
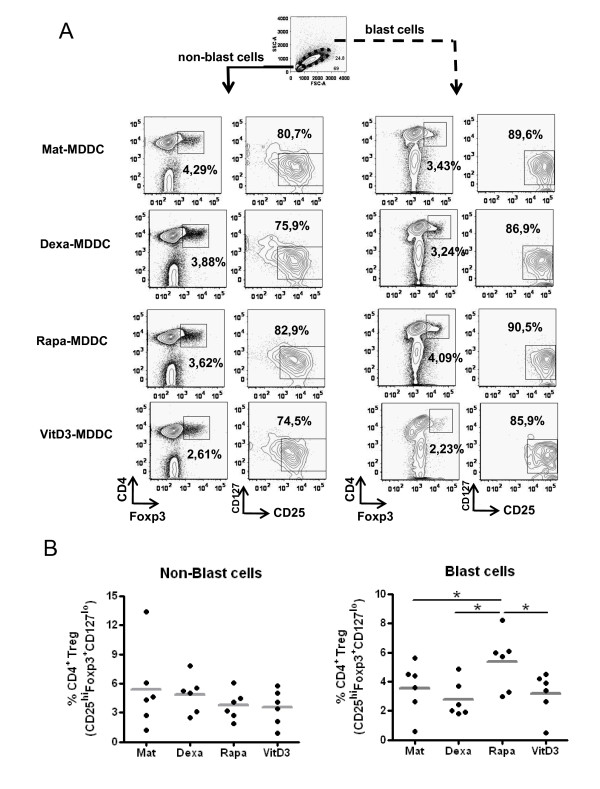
**Rapa-DCs promote CD4+CD25^hi^CD127^low^FoxP3+ induction from blast T cells**. After 6 days of culture without re-stimulation and any supplemental cytokines, cell sizes were evaluated by FACS by plotting forward scatter (FSC) versus side scatter (SSC) parameters. Small (solid line) non-blast cells and large (dotted line) blast cells are circled. (A) Phenotype of T cells as CD4+, Foxp3+ and CD25+ with low or null CD127 expression. One of 6 representative experiments is shown. (B) Summary of percentages of T cells in non-blast (left) and blast (right) cells. (* p ≤ 0.05, n = 6, paired t-test).

Finally, the presence of Tregs cells defined as CD4+ CD127 low/negative CD25high and Foxp3+ as reported before (72) was estimated in these culture conditions. After one round of stimulation for 6 days, we analysed the induction of CD4+ Foxp3+ and CD25^high^, CD127^low/negative ^cells as shown in Figure 6A. Then, as depicted, only those T cells stimulated by Rapa-DCs showed a significantly increase of the percentages of CD4+ Foxp3+ and CD25^high^, CD127^low/negative ^cells (5.4 ± 1.9% vs. 3.5 ± 1.7% with Mat-DCs, p = 0.0211, n = 6, paired t test) (Figure 6B).

## Discussion

Induction of therapeutic tolerance is of increasing interest in autoimmunity, allograft rejection, allergy, asthma, and various forms of hypersensitivity. Because of their capacity to orchestrate immune responses, DCs can be used as therapeutic agents. The classical concept that immature DCs induce tolerance and that mature DCs induce immune responses has changed completely, and several lines of evidence demonstrate that the maturation state of DCs does not always correlate with their tolerising or activating functions [43]. In this sense, the definition of tol-DCs must include a maturation-resistant cell that acts as "an immature DC" with a stable phenotype that is preserved, even in the presence of pro-inflammatory signals. This tolerogenic state of DCs can be induced using several pharmacological agents [44-46].

At present, scattered knowledge from different tolerogenic cellular products can be found. A better understanding of clinical grade cellular therapies may offer new opportunities for treating different disorders. However, several gaps in our knowledge remain to be filled-in before a perfect tolerogenic DC (one best suited for targeting a particular process) may be envisaged. Thus, our work aimed to determine the capabilities of those GMP-grade immunosuppressive drugs (dexamethasone, rapamycin and vitamin D3) that are used to obtain tol-DCs in comparative scenarios and identify the "array" of their individual characteristics, such as phenotypes, cytokine profiles, resistance to maturation, and T-cell profiles, in order to define the best DCs for a particular situation.

Hence, we report for the first time a comparative study of clinical-grade tolerogenic cellular products for therapeutic applications that fulfil the regulatory medical rules for human therapy. Our results show that all clinical-grade tol-DCs that were analysed function as "negative cellular vaccines," which are comparable to previously characterised research-grade tol-DCs [47]. In terms of viability, we observed that VitD3 had a slight tendency to promote DC apoptosis, in accordance with previous reports [48]. However, this minor reduction in cell viability does not compromise either DC functionality or the eventual use of these cells in therapy. Although apoptosis induction in DCs by pharmacological agents has been controversial, several reports demonstrated that Dexa did not induce cell death in MDDCs at any of the tested concentrations [49,50]. Also, use of Rapa for DC maturation did not increase apoptosis [51], in agreement with our results.

When analysing the phenotypes of the generated tol-DCs, we observed that only Dexa-and VitD3-DCs had reduced classical markers of mature cells on their surfaces. However, Rapa-DCs did not show an immature phenotype, thus being characterized as "mature DCs" with respect to their exhibited phenotype. In this context, it is obvious that the definition of DC maturation using phenotype markers is not a distinguishing feature of immunogenicity nor tolerogenicity [40]. Thus, a set of "biomarkers" for tolerance induction in our cellular products have to be defined to better monitor the putative tolerogenic cells [17,37], as phenotypic identification of tol-DCs may not be as accurate as expected. Ideally, quality controls for tol-DCs should be based on markers that are quickly and readily detectable and that are reliable.

From the cytokine profile results, Dexa-and moderately VitD3-derived DCs showed increased IL-10 production, whereas the secretion of IL-12p70 was not detected in all cases. It is well known that IL-10 blocks IL-12 synthesis by DCs, downregulates the expression of co-stimulatory molecules and potentiates their tolerogenicity [43,52]. This tolerogenic feature was not observed with Rapa-DCs, as was previously reported [53]. Most likely, DCs modified by Rapa use some other mechanism to induce tolerance, as discussed below.

Resistance to maturation is considered a prerequisite of tolerogenic potential for ''negative cellular vaccines''. Under the influence of inflammation, the administered immature DCs should potentially undergo maturation and lose their tolerogenic function. Thus, for good clinical applications, tol-DCs should show a stable immunosuppressive phenotype that will not be transformed to immunostimulatory DCs after injection into patients. In this context, several methods have been described for designing maturation-resistant DCs [54-57]. Our results show that Dexa-DCs, and to a lesser extent VitD3-DCs, exhibit a durable "immaturity," as high IL-10 production and no IL-12/IL-23 production was maintained upon subsequent TLR stimulation. In agreement with this, Xia et al. previously demonstrated that this tolerogenic product preserves this feature up to 5 days after removing Dexa [58]. As described in the literature, immature DCs undergo maturation and lose their tolerogenic functions. Interestingly, the cytokine profiles of the generated tol-DCs were not modified by a strong TLR stimulation, indicating that they maintained a stable profile.

Another functional property of tol-DCs is their decreased T cell-stimulatory capability. We further investigated the immunoregulatory capability of clinical-grade tol-DCs using direct T cell activation in mixed-lymphocyte reactions. Our results showed differential potentials for reducing proliferation: Rapa and VitD3 worked in the nM range, while Dexa required higher concentrations in the μM range. In fact, tolerogenic MDDCs conditioned with Dexa from 1/3 of the individuals (4/12) did not acquire regulatory properties at the concentration used, and even showed a "semi-mature" phenotype. In this regard, the possibility of combining Dexa with VitD3 to prevent de-sensitization of the DCs to the actions of Dexa has been reported [11]. Furthermore, both immunomodulatory agents used in combination inhibit DC maturation and function in an additive manner [7,59,60].

In addition, total IFN-γ production was significantly reduced when these T cells were stimulated by tol-DCs. To extend our analyses, we evaluated IFN-γ in T cells that had responded to allostimulation and observed that IFN-γ production was only reduced when Rapa-DCs were used as stimulators. This property in the deviation of Th differentiation was also observed previously by Monti P. et al [61].

It has been described that tolerogenic DCs induce immune tolerance through several pathways, including clonal T cell depletion or exhaustion, anergy, deviation of Th differentiation or generation of Tregs [15,62-68]. To deduce which mechanisms that tol-DCs might have exerted, the possibility of apoptosis induction was evaluated. However, we did not find any differences in cell death by allostimulated T cells, indicating that this mechanism was not acting in our cellular products. In contrast, it has been reported that Dexa-and VitD3-DCs induced a hyporesponsiveness as a strategy to dampen autoreactive responses [50], and our own observations (Raïch-Regué D. et al) support these results.

Finally, we tested for the induction of CD4+CD25^hi^CD127^low^FoxP3+ T cells. Regulatory T cells suppress the responses of alloreactive or self-reactive CD4+ T cells and are supposed to maintain immunologic self-tolerance or control autoimmunity [69-71]. Rapa-DC-primed T cells exhibited reduced alloproliferation along with a concomitant expansion of CD4+CD25^hi^CD127^low^FoxP3+ cells [72-74]. This effect may have been in response to the expression of high levels of CD86 and is consistent with previous reports that described that co-stimulation is required for induction and expansion of FoxP3+ Tregs [53,75,76]. In contrast, Dexa and VitD3 did not induce this phenotype on T cells. This discrepancy with the literature could be due to the particular experimental approaches. It is important to note that we analyzed these T cells in co-cultures of MDDCs with allogenic T cells for one round of stimulation. However, it has been demonstrated that VitD3-DCs convert naive T cells into Tregs after several rounds of priming and boosting [77]. Another possibility to explore was the presence of other CD4+ Treg subsets, including CD4+CD25-FoxP3-IL-10 producing Tr1 cells [78,79] and transforming growth factor-β (TGF-β+) Th3 cells [80]. In this sense, our results show IL-10 production on T cells stimulated by Dexa-DCs but not TGF-β in any of cultured conditions.

## Conclusions

In summary, in these comparative analyses of clinical grade tol-DCs, Dexa-and VitD3-DCs exhibited a "semi-immature" phenotype and IL-10 secretion. In contrast, Rapa-DCs induced CD4+CD25^hi^CD127^low^FoxP3+ and inhibited IFN-γ secretion by allostimulated T cells. This comparative study emphasises the fact that a simple phenotypic determination of maturation markers does not guarantee a tolerogenic function and that a complete set of functional determinations is mandatory in order to clearly define a tolerogenic "functional" phenotype. This also stresses the necessity to define reliable biomarkers for applications in GMP labs. Finally, this may also help with decisions on which tolerogenic product will be the best for a particular situation. Phase I-II studies with quality control measures and appropriate clinical and immunological outcomes must be performed to evaluate potential tol-DC functions.

## List of abbreviations

DC: dendritic cell; Dexa: dexamethasone; GMP: Good Manufacturing Practice; IFN-γ: Interferon-gamma; Io: ionomycin; MDDC: Monocyte Derived DC; PBMCs: Peripheral Blood Mononuclear Cells; PMA: phorbol 12-myristate 13-acetate; Rapa: rapamycin; tol-DC: tolerogenic DCs; Tregs: regulatory T cells; VitD3: vitamin D3.

## Authors' contributions

MNG conceived and designed the study, performed most of the experiments and drafted the manuscript. DRR carried out the immunophenotyping and the determination of Tregs, participated in the design of the study and helped in writing the manuscript. CO contributed in cell culture techniques and analysed data. LGL participated in the statistical analysis and interpretation of data. CR participated in the analysis and revised the manuscript. RPB, head of the lab, critically revised the manuscript. EMC participated in the coordination of the study and helped to draft manuscript. FEB, author for correspondence, participated in the design of the study, supervised the research, and revised the manuscript. All authors read and approved the final manuscript.

## Competing interests

The authors declare that they have no competing interests.

## Supplementary Material

Additional file 1 **Figure S1-Dose-dependent experiments to establish equivalent tol-DCs**. Summary of the dose-dependent experiments set up to obtain the optimal concentration of each immunomodulatory agent. The results reflected the relative values of the alloproliferation of T cells co-cultured with different tol-DCs (A: Dexa-DCs, n ≥ 2; B: Rapa-DCs, n = 3; C: VitD3-DCs, n = 4). Click here for file
