# Supplementary figures and images for: Comparative study of clinical grade human tolerogenic dendritic cells
Source: J Transl Med. 2011 Jun 9;9:89. doi: 10.1186/1479-5876-9-89 (PMC3141500; doi:10.1186/1479-5876-9-89)

## Slide 1
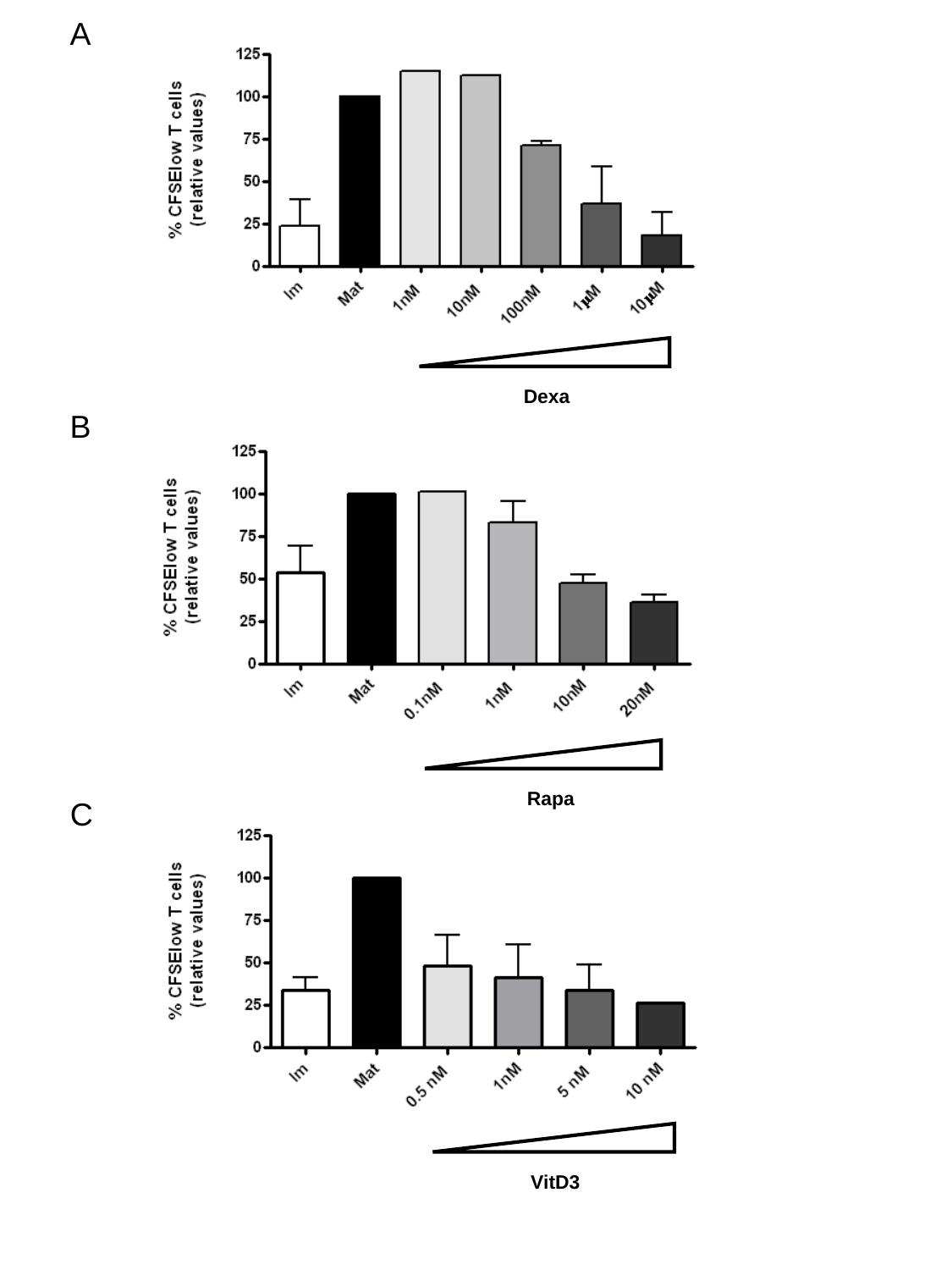

A
Dexa
B
Rapa
C
VitD3

Supplement: Additional file 1 — Figure S1-Dose-dependent experiments to establish equivalent tol-DCs. Summary of the dose-dependent experiments set up to obtain the optimal concentration of each immunomodulatory agent. The results reflected the relative values of the alloproliferation of T cells co-cultured with different tol-DCs (A: Dexa-DCs, n ≥ 2; B: Rapa-DCs, n = 3; C: VitD3-DCs, n = 4). [file 1479-5876-9-89-S1.PPT]
